# Tiletamine/Zolazepam and Ketamine with Dexmedetomidine (TKD) Cocktail Is as Effective as Tiletamine/Zolazepam and Ketamine with Xylazine (TKX) in Providing Pig General Anesthesia

**DOI:** 10.3390/ani14192881

**Published:** 2024-10-07

**Authors:** Ekkapol Akaraphutiporn, Sumit Durongphongtorn, Katechan Jampachaisri, Patrick Sharp, Cholawat Pacharinsak, Chalika Wangdee

**Affiliations:** 1 Department of Veterinary Surgery, Faculty of Veterinary Science, Chulalongkorn University, Bangkok 10330, Thailand; ekkapol.a@chula.ac.th (E.A.); sumit.d@chula.ac.th (S.D.); 2 Department of Mathematics, Faculty of Science, Naresuan University, Phitsanulok 65000, Thailand; katechanj@nu.ac.th; 3 Department of Animal Research Services, University of California Merced, Merced, CA 95343, USA; patricksharp@hotmail.com; 4 School of Veterinary and Life Sciences, Murdoch University, Perth, WA 6150, Australia; 5 Department of Comparative Medicine, Stanford University, Stanford, CA 94305, USA; cholawat@stanford.edu; 6 Center of Excellence in Biomaterial Engineering in Medical and Health, Chulalongkorn University, Bangkok 10330, Thailand

**Keywords:** anesthesia, dexmedetomidine, pig, tiletamine/zolazepam, xylazine

## Abstract

**Simple Summary:**

In this study, we evaluate dexmedetomidine as a potential alternative to xylazine for providing general anesthesia in pigs. Xylazine, a commonly used alpha-2 (α-2) adrenergic agonist, provides sedative, muscle relaxant, and analgesic effects with rapid onset but is often associated with side effects such as bradycardia, respiratory depression, and hypotension, which can limit its use. Dexmedetomidine, a more selective α-2 agonist, is gaining popularity in veterinary medicine for its enhanced sedation and analgesia, despite similar risks of cardiorespiratory suppression. We proposed that combining dexmedetomidine with tiletamine/zolazepam and ketamine (TKD) could reduce drug dosages, potentially minimizing side effects. While tiletamine/zolazepam, ketamine, and xylazine (TKX) is widely used in pigs, research on TKD is limited. This study aimed to compare the anesthetic efficacy of TKD with TKX in pigs undergoing short-term (45-min) and long-term (90-min) surgeries, hypothesizing that TKD would provide anesthesia comparable to, or even better than, TKX for both durations.

**Abstract:**

This study aimed to evaluate dexmedetomidine as an alternative to xylazine in pigs. We compared TKD (0.05 mL/kg) to TKX (0.05 mL/kg) in 20 male pigs undergoing unilateral cryptorchid castration (short-term, 45-min) or bilateral cryptorchid castration (long-term, 90-min). We hypothesized that TKD would be comparable to TKX for both short-term and long-term anesthesia. Monitored parameters were classified into duration and physiological categories, including induction and recovery times, reflexes, heart rate (HR), respiratory rate (RR), arterial blood pressure, oxygen saturation (%SpO2), end-tidal carbon dioxide (ETCO2), and body temperature (TEMP). Isoflurane levels were also recorded, if used. Results showed no significant differences in duration parameters between TKD and TKX for either short-term or long-term anesthesia (induction: 1 min; recovery: 18–35 min). Physiological parameters were mostly similar between groups, although TKD caused slightly higher blood pressure during short-term anesthesia. Isoflurane levels (0.1–0.6%) were comparable between groups. Overall, the results suggest that TKD provides anesthesia comparable to TKX in pigs undergoing unilateral or bilateral cryptorchid surgery requiring short-term and long-term anesthesia.

## 1. Introduction

To anesthetize pigs, a combination of injectable anesthesia with or without isoflurane is commonly used depending on the procedure’s duration. Examples of injectable anesthetics include tiletamine/zolazepam or ketamine with sedatives i.e., xylazine [1,2,3,4]. Although ketamine alone works well in this species, ketamine supplemented with tiletamine/zolazepam (Telazol or Zoletil) has gained momentum. Tiletamine/zolazepam, a nonnarcotic and nonbarbiturate combination, is commercialized as an equal weight combination powder of two drugs: tiletamine hydrochloride, a non-competitive N-methyl-d-aspartate (NMDA) receptor antagonist, and zolazepam hydrochloride, a water-soluble benzodiazepine sedative [5,6]. While zolazepam and tiletamine individually may have adverse central nervous system (CNS) effects, when combined the side effects are significantly reduced [5,7]. The advantages of a tiletamine/zolazepam combination include tiletamine’s rapid onset of action, anesthesia, and some analgesia, while zolazepam provides good sedation, anxiolysis, and muscle relaxation [5,8]. Tiletamine is more potent and has a longer duration of action than ketamine. Regarding the similarities between ketamine and tiletamine/zolazepam, combining these two drugs could reduce the dosage of each and lessen any individual adverse effects [9]. According to previous studies, using ketamine as the sole agent caused tachycardia, hypertension, unwanted CNS effects, and poor muscle relaxation [5,8].

Xylazine and dexmedetomidine are commonly used sedatives in research pigs. These alpha-2 (α-2) receptor agonists provide muscle relaxation, sedation, and analgesia, and are usually combined with other compounds i.e., ketamine to provide neuroleptanalgesia as a premedication or an anesthetic [8,9,10]. Conversely, a-2 adrenergic agonists provide dose-dependent sedation and suppress cardiorespiratory systems resulting in bradycardia, hypertension/hypotension, and respiratory depression [10,11]. A tiletamine/zolazepam and dexmedetomidine combination with or without tramadol provided a surgical anesthesia plane in male and female Sprague–Dawley rats for 45 min [12,13]. Pigs with tiletamine/zolazepam alone (4.4 mg/kg) or tiletamine/zolazepam with ketamine (4.4 mg/kg and 2.2 mg/kg, respectively) could not be intubated. Intramuscular (IM) tiletamine/zolazepam and xylazine (4.4 and 2.2 mg/kg, respectively) or IM tiletamine/zolazepam, ketamine, and xylazine (4.4, 2.2 and 2.2 mg/kg, respectively) provided lateral recumbency (approximately 45–59 min and 50–72 min, respectively) and analgesia (approximately 18–40 min and 23–48 min, respectively) [3].

Although xylazine is commonly used, the more specific α-2 receptor agonist, dexmedetomidine, has gained popularity in veterinary medicine over the last decade [11,14,15,16]. Dexmedetomidine is a highly selective α-2 adrenergic agonist, which works by binding to α-2 receptors in the CNS. Upon binding, it reduces the release of norepinephrine through a presynaptic negative feedback mechanism. This leads to decreased sympathetic outflow, which results in sedation, anxiolysis, and analgesia. The inhibition of norepinephrine release prevents the transmission of pain signals and suppresses neuronal firing in pathways responsible for consciousness and nociception [17,18]. Using the concept of neuroleptanalgesia, combining dexmedetomidine with tiletamine/zolazepam and ketamine, lower dosages of each agent can be used leading to fewer side effects overall. Although the TKX combination has been used in pigs, little is known about TKD.

Therefore, this current study investigated the anesthetic effects of these two combinations in two different conditions. The objective was to evaluate general anesthesia induced by either TKD and TKX in pigs undergoing surgery for short-term (45-min) and long-term (90-min) anesthesia. We hypothesized TKD-induced general anesthesia would be comparable to TKX for both short- and long-term pig anesthesia.

## 2. Materials and Methods

### 2.1. Animals

Twenty male, intact crossbred pigs (Duroc × Large White Yorkshire × Landrace) with an average age of 5.2 ± 2.0 weeks and 15.6 ± 8.6 kg body weight were obtained from Betagro Farm, Thailand. Pigs were housed four per conventional pen (6 m^2^) during the acclimatization period. Pigs had ad libitum access to a standard commercial diet (Betagro 302, Betagro, Thailand) and drinking water. Prior to anesthetic induction, food and water were withheld for at least 12 and 6 h, respectively. Physical examination results and hematologic analysis (complete blood count and blood chemistry profiles) were within normal ranges for healthy pigs. All pigs had either unilateral or bilateral cryptorchidism and were scheduled for surgical castration. This study was reviewed and approved by the Animal Care and Use Committee, Faculty of Veterinary Science, Chulalongkorn University.

### 2.2. Experimental Design

In this study, twenty pigs were divided into two groups depending on their cryptorchidism, unilateral (*n* = 10) and bilateral (*n* = 10), which affected the surgical and anesthetic time. The unilateral cases required short-term anesthesia (45-min) and bilateral cases required long-term anesthesia (90-min). Pigs in each group were further randomly assigned into two other treatment groups comparing anesthesia induced by TKD or TKX. In total, each group consisted of five pigs with unilateral cryptorchidism and five pigs with bilateral cryptorchidism.

Regarding the drug combinations used in this study, first, tiletamine/zolazepam powder (Zoletil, Virbac, Thailand; 250 mg tiletamine and 250 mg zolazepam) was diluted with 2.5 mL of ketamine (Ketamine, Alfasan Nederland BV, Netherlands; 100 mg/mL) to formulate the tiletamine/zolazepam and ketamine combination. To reconstitute the TKD formulation, 2.5 mL of dexmedetomidine (Dexdomitor, Virbac, Thailand; 0.5 mg/mL) was added. To reconstitute the TKX formulation, 2.5 mL of xylazine (X-Lazine, L.B.S. Laboratory Ltd., Thailand; 100 mg/mL) was added. Either TKD or TKX was administered IM as 0.05 mL/kg, which corresponded to 5 mg/kg tiletamine/zolazepam/2.5 mg/kg ketamine/0.0125 mg/kg dexmedetomidine for TKD and 5 mg/kg tiletamine/zolazepam/2.5 mg/kg ketamine/2.5 mg/kg xylazine for TKX. The injection site was the dorsolateral neck muscles and administered using an 18 G x 1” hypodermic needle (Nipro, Nipro Corporation Ltd., Bangkok, Thailand).

### 2.3. Anesthesia and Surgical Procedure

Once the appropriate anesthetic effects were achieved, indicated by lateral recumbency followed by the loss of jaw tone and palpebral reflex, the auricular vein was catheterized and normal saline solution (NSS, General Hospital Products (Public) Co., Ltd., Bangkok, Thailand) was administered (5–10 mL/kg/h). Endotracheal intubation was performed by a single investigator (CW), while slow intravenous (IV) propofol (Propofol-Lipuro, B. Braun Melsungen AG, Melsungen, Germany; 10 mg/mL) was administered as needed. Body temperature was maintained by warm water blanket (T Care Circulating Thermal Water System, KimuraMed, Tokyo, Japan) through anesthetic recovery. An appropriate surgical plane was maintained by isoflurane (Attane, Piramal Critical Care, Bethlehem, PA, USA) inhalation with 100% oxygen. Briefly, isoflurane levels were adjusted based on reflexes, respiratory effort, and the pigs’ responses to surgical stimuli. The isoflurane concentration used in this study was recorded throughout the anesthetic procedure.

Prior to the surgery, 25 mg/kg cefazolin (Cefamezin, Biolab Co., Ltd., Bangkok, Thailand) was administered via IV as a prophylactic antibiotic. Pigs were positioned in dorsal recumbency, followed by hair clipping and surgical scrub of the surgical site. A lidocaine (4 mg/kg, 20 mg/mL Locana, L.B.S. Laboratory Ltd., Bangkok, Thailand;) line block along the incision line provided local anesthesia. For the unilateral cryptorchism, the testis in the scrotal sac was removed via an incision overlying the testis. The testis was then carefully removed from the scrotal sac with its intact vaginal tunic, then the spermatic cord and vaginal tunic were ligated with an absorbable suture material. After ligation, the spermatic cord was clamped with arterial forceps and cut with a scalpel, while bleeding was carefully observed after the arterial forceps were removed. The surgical wound was left open and povidone-iodine was applied around the surgical site. The cryptorchid technique consisted of a paramedian approach performed by making an incision lateral to the prepuce on the side of the cryptorchid testis. The rectus abdominis muscle and peritoneum were incised, followed by blunt dissection as needed. The retained testis was carefully pulled out, ligated, clamped, and removed. A routine abdominal wall closure was performed using the appropriate suture material, followed by povidone-iodine application to the surgical wound. The surgery was performed by a veterinary student under the close supervision of a certified veterinarian.

Following surgery, 0.32 mg/kg atipamezole (Antisedan, Virbac, Bangkok, Thailand; 5 mg/mL) was administered IM to reverse either dexmedetomidine or xylazine. After the endotracheal tube removal, pigs were transferred to a recovery pen and monitored by a certified veterinarian until ambulatory.

### 2.4. Anesthetic Monitoring and Assessments

Duration parameters: The duration parameters observed in this study were classified into two categories: the induction duration and the recovery duration. The induction duration was defined as the interval between the TKD or TKX injection and the clinical response to the anesthetic drugs. The clinical responses observed during the induction phase were as follows: (1) sternal recumbency; (2) lateral recumbency; (3) loss of withdrawal reflex; (4) loss of jaw tone; and (5) loss of palpebral reflex. The recovery duration was defined as the interval between the atipamezole injection and the clinical responses during recovery phase, including: (1) return of palpebral reflex; (2) return of jaw tone; (3) return of withdrawal reflex; (4) return of sternal recumbency; (5) ability to stand; and (6) ability to walk unassisted.

Physiological parameters: Once intubated, the pigs were monitored and the results were recorded every 5 min throughout anesthesia as related to the following physiological parameters: body temperature (TEMP), oxygen saturation (%SpO2), end-tidal carbon dioxide (ETCO2), heart rate (HR), respiratory rate (RR), systolic, mean, and diastolic arterial blood pressure (SAP, MAP, and DAP, respectively) using anesthetic monitoring devices (Comen C80 Multi-Parameter Patient Monitor, Shenzhen Comen Medical Instrument Co., Ltd., Shenzhen, China) and capnometer (CapnoEasy, Beijing Winland Medical Co., Ltd., Beijing, China).

### 2.5. Statistical Analysis

The duration parameters and isoflurane concentration were analyzed using the Mann–Whitney U test, and these data were reported as median ± interquartile range (IQR). The physiological parameters were compared by using the ANOVA test followed by the Bonferroni multiple comparison test. These data were reported as mean ± standard error of the mean (SEM). A *p*-value of less than 0.05 was considered statistically significant. Data analysis was performed using SPSS 28.0 software (SPSS Inc., IBM Corp, Armonk, NY, USA).

## 3. Results

### 3.1. Duration Parameters

Induction: Out of the 10 pigs induced with either TKD or TKX in each group, 3 pigs (30%) in the TKD group required propofol at a dose of 0.50 (0.45–0.50) mg/kg for endotracheal intubation, while 2 pigs (20%) in the TKX group needed propofol at 0.76 (0.63–0.88) mg/kg. The time to sternal (1.14 (1.05–1.35) and 1.04 (0.79–1.55) min, respectively) and lateral (1.82 (1.49–2.36) and 1.59 (1.40–2.16) min, respectively) recumbency for TKD or TKX showed no significant differences. Similarly, the time to loss of withdrawal reflex (2.90 (2.55–6.80) and 3.68 (3.36–4.70) min, respectively), jaw tone (5.04 (2.71–7.55) and 4.03 (3.55–5.13) min, respectively), and palpebral reflex (5.75 (3.62–9.99) and 4.85 (4.28–7.88) min, respectively) were not significantly different between TKD and TKX (Table 1).

Recovery (short-term anesthesia, 45-min): All recovery parameters were monitored after atipamezole administration. The return of palpebral reflex (3.0 (2.0–9.0) and 7.0 (5.0–13.0) min, respectively), jaw tone (10.0 (3.0–14.0) and 6.0 (6.0–8.0) min, respectively), withdrawal reflex (12.0 (7.0–24.0) and 11.0 (6.0–19.0) min, respectively), and sternal recumbency (18.0 (8.0–39.0) and 35.0 (25.0–35.0) min, respectively) were not significantly different between TKD and TKX (Table 2). Similarly, the time to stand (84.0 (71.0–88.0) and 81.0 (69.0–171.0) min, respectively) and to walk (84.0 (74.0–110.0) and 87.0 (81.0–188.0) min, respectively) were not significantly different between TKD and TKX.

Recovery (long-term anesthesia, 90-min): All recovery parameters were monitored after atipamezole administration. The return of palpebral reflex (2.0 (2.0–8.0) and 15 (8.0–16.0) min, respectively), jaw tone (7.0 (3.0–9.0) and 7.0 (7.0–10.0) min, respectively), withdrawal reflex (10.0 (3.0–14.0) and 17.0 (12.0–20.0) min, respectively), and sternal recumbency (18.0 (17.0–32.0) and 28.0 (17.0–100.0) min, respectively) were not significantly different between TKD and TKX groups (Table 2). As was seen with short-term anesthesia, the time to stand (39.0 (26.0–96.0) and 147.0 (100.0–221.0) min, respectively) and to walk (58.0 (37.0–97.0) and 149.0 (101.0–223.0) min, respectively) were not significantly different between TKD and TKX.

### 3.2. Physiological Parameters

**Figure 1:**
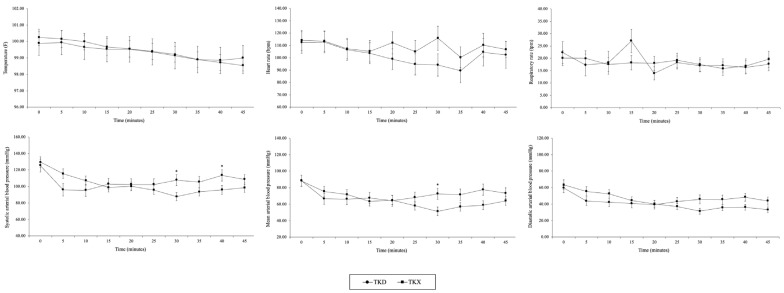
Physiological parameters, including temperature (TEMP), heart rate (HR), respiratory rate (RR), systolic, mean, and diastolic arterial blood pressure (SAP, MAP, and DAP, respectively), are presented as mean values ± standard error of the mean (SEM) for the TKD and TKX groups within short-term anesthesia (45-min). (compared between group: * *p* < 0.05).

Short-term anesthesia: The %SpO2 and ETCO2 were maintained within the normal range, with %SpO2 remaining above 95% and ETCO2 within 35–45 mmHg. Other physiological parameters, such as TEMP, HR, RR, and DAP, showed no significant differences between the groups. However, MAP was significantly higher in the TKD group (72.6 ± 6.8) compared to the TKX group (51.3 ± 5.2) at 30-min (*p* = 0.021). In addition, SAP was significantly higher in the TKD group compared to the TKX group at 30-min (102.5 ± 6.8 vs. 87.7 ± 4.8, respectively (*p* = 0.019)) and at 40-min (113.5 ± 6.9 vs. 96.0 ± 5.3, respectively (*p* = 0.041)) (Figure 1).

**Figure 2:**
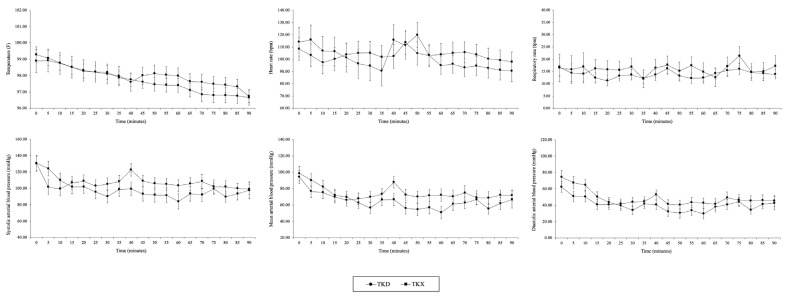
Physiological parameters, including temperature (TEMP), heart rate (HR), respiratory rate (RR), systolic, mean, and diastolic arterial blood pressure (SAP, MAP, and DAP, respectively), are presented as mean values ± standard error of the mean (SEM) for the TKD and TKX groups within long-term anesthesia (90-min).

Long-term anesthesia: Similarly, %SpO2 and ETCO2 were consistently maintained within normal ranges (above 95% for %SpO2 and 35–45 mmHg for ETCO2), while all observed physiological parameters (TEMP, HR, RR, SAP, MAP, and DAP) were not significantly different over time (Figure 2).

### 3.3. Isoflurane Concentration

For the short-term anesthesia, the isoflurane concentration did not significantly different between TKD (0.18% (0.18–0.65)) and TKX (0.60% (0.33–1.00)). Similarly, for long-term anesthesia, there was no significant difference in the isoflurane concentration between TKD (0.45% (0.39–0.54)) and TKX (0.16% (0.12–0.37)) (Table 3).

## 4. Discussion

Our study demonstrated that TKD and TKX provided comparable general anesthesia during short- and long-term anesthesia. Our findings include: (1) for duration parameters, there were no significant differences between TKD and TKX for short and long-term anesthesia; (2) for physiological parameters, during short-term anesthesia, MAP (at T30) and SAP (at T30 and T40) were significantly higher with TKD vs. TKX. Other physiological parameters were comparable for short- and long-term anesthesia; (3) there was no significant difference in isoflurane levels between both groups during short- and long-term anesthesia.

This study’s objective was to compare general anesthesia induced by either TKD or TKX in short- and long-term anesthesia from the patient’s induction to recovery. TKD or TKX was demonstrated to be effective for general anesthesia for noninvasive imaging [19]; here, we further investigated TKD’s or TKX’s general anesthesia effectiveness in a pig surgical model necessitating short- and long-term anesthesia. We chose similar TKD and TKX doses previously published in the noninvasive imaging, as pigs were more than effectively maintained during imaging in that study [19]. The authors expected similar TKD and TKX doses would provide sufficient general anesthesia in these surgical models requiring short- and long-term anesthesia. However, in this current study, which included a surgical procedure, if a deeper anesthetic plane was required, we were prepared to add isoflurane, when needed.

Induction—There were no significant differences between TKD and TKX. Sternal recumbency was achieved at 1 min with lateral recumbency occurring between 1.5–1.8 min. The studied combination achieved rapid sternal and lateral recumbency onset similar to tiletamine/zolazepam (4.4 mg/kg) alone, tiletamine/zolazepam (4.4 mg/kg) with xylazine (2.2 mg/kg), and tiletamine/zolazepam (4.4 mg/kg) with ketamine (2.2 mg/kg) and xylazine (2.2 mg/kg) [3]. This study showed the loss of withdrawal, jaw tone, and palpebral reflexes occurred within 5 min. After the loss of jaw tone and palpebral reflex (within 5 min), 30% of pigs in the TKD group and 20% in the TKX group required propofol for successful intubation (without coughing or chewing during intubation), while the remaining pigs did not require propofol. Although propofol was used as an adjunct induction agent in some pigs in this study, other studies have demonstrated successful pig intubation within 5 min of anesthetic (TKX) administration without the need for additional induction agents [3].

Recovery—For short-term anesthesia, there were no significant differences between TKD and TKX. Return of palpebral reflex (3–7 min), jaw tone (6–10 min), and the withdrawal reflex (11–12 min) were similar. Pigs regained sternal recumbency at 18–35 min, stood at 81–84 min, and were walking at 84–87 min. Similar results occurred in the long-term anesthesia cohort; there were no significant differences between TKD and TKX. The return of the palpebral reflex (2–15 min), jaw tone (7 min), and the withdrawal reflex (10–17 min) were similar. Pigs obtained sternal recumbency at 18–28 min, stood at 39–147 min, and were walking at 58–149 min. Studies showed that when α-2 antagonists were used, the recovery time was shortened [12,19]. It is important to note this current study administered atipamezole at the end of the procedure to reverse either dexmedetomidine or xylazine. Other studies showed that tolazoline (2 mg/kg IM, α-2 antagonist) reversed detomidine in a 45-min pig anesthesia (3 mg/kg tiletamine/zolazepam, 0.18 mg/kg detomidine, and 0.12 mg/kg butorphanol). Although the recovery time was shortened, the quality of anesthetic recovery was not diminished [1]. Due to dexmedetomidine’s longer duration of action (vs. xylazine), failure to use atipamezole may result in longer recovery times with TKD vs. TKX [20,21]. Zolazepam’s plasma half-life varies by species: 1–4 h (dogs (4 h), nonhuman primates (1 h), cats (4.5 h), and rats (3 h)) [6,7,10]. In domestic pigs, tiletamine’s elimination time and half-life (0.2 h and 3 h, respectively) was faster than that of zolazepam (0.1 h and 8 h, respectively) [6,9]. Therefore, the authors decided not to reverse zolazepam with flumazenil, a benzodiazepine antagonist. In addition, it is important to note that flumazenil may have species-specific effects. For example, flumazenil did not improve or hasten recovery in cats (3 mg/kg ketamine and 0.05 mg/kg midazolam) [10,22]. By contrast, flumazenil (0.08 mg/kg IV) successfully shortened the tiletamine/zolazepam (4.4 mg/kg, IM) recovery time in pigs [8,23]. It is vitally important to note that atipamezole reverses both sedation and analgesia. Therefore, before administering atipamezole, one must administer other analgesic classes and they must be at effective levels. In our study, lidocaine was locally administered into the testicles. Although this study did not measure the analgesic level, we did use the loss of the paw withdrawal reflex to show sufficient surgical anesthesia with the minimal addition of isoflurane [5,24,25].

Isoflurane-for both short- and long-term anesthesia, additional isoflurane (<1%) was needed for all pigs. These results indicated the TKD and TKX dosages were insufficient to maintain general anesthesia involving surgical manipulation. A previous study demonstrated TKD and TKX dosages were sufficient to maintain a lighter plane of general anesthesia for non-surgical manipulation (imaging) [19]. Because higher tiletamine/zolazepam doses may yield a prolonged recovery, to prevent a prolonged recovery, the authors chose to use similar limited doses as were used in a previous imaging study [19,26]. It is possible that the addition of an opiate or a higher tiletamine/zolazepam dose in this study could have elicited a sufficiently deeper anesthetic plane without the need for isoflurane [4,26,27].

In this study, physiological parameters were not significantly different throughout the study for either short- or long-term anesthesia. This was similar to a report in monkeys (*Macaca facicularis*) where tiletamine/zolazepam alone resulted in no change in blood gas parameters [28]. The only physiological change reported in the monkey study was decreased body temperature. Dexmedetomidine, known for its cardiovascular effects, acts as a more selective α-2 adrenergic agonist, causing an initial increase in blood pressure due to peripheral vasoconstriction [16,29,30]. However, in our study, significant differences in arterial blood pressure between the TKD and TKX groups were noted at only two time points, suggesting that the cardiovascular impact of dexmedetomidine was transient and not clinically significant overall. While body temperature differences were not statistically significant, there was a trend of decreasing body temperature over time, especially in the long-term pig anesthesia cohort. This underscores the importance of keeping any patient warm, as decreased body temperature may cause prolonged anesthetic recovery [31,32]. A study in horses (IV, tiletamine/zolazepam with ketamine and detomidine) also showed no change in physiological parameters (cardiorespiratory, acid-base status, electrolytes, and hematologic values), with the quality of anesthesia and analgesia reported as satisfactory [33].

In providing balanced anesthesia, adding ketamine and dexmedetomidine to tiletamine/zolazepam decreases the volume and elicits a greater potency of all anesthetics [3,34]. This study showed tiletamine/zolazepam worked well in pigs; however, in cats, hyperthermia (106 F or 41 C) with a restlessness recovery (with head bobbing) may develop with tiletamine/zolazepam (4.0–4.4 mg/kg, IM) [35]. Tiletamine/zolazepam is clinically contraindicated in pancreatitis, renal disease, or feline urethral obstruction [5,35]. Although postanesthetic pulmonary edema was reported in cats with tiletamine/zolazepam use, we did not clinically observe these side effects in pigs [35].

This study has several limitations. (1) Only male pigs were studied. As the first study using this surgical model, only male pigs were studied to create uniformity in the sample. The inclusion of female pigs might have yielded different results [36]. Therefore, only males were studied. (2) Analgesia levels were not measured. The authors only used non-movement to surgical manipulation as an indicator for achieving a sufficient surgical anesthesia plane. (3) Other analgesics (NSAIDs) were not used. The authors mimicked actual farm clinical practice where lidocaine local administration is often performed. (4) Only a single TKD or TKX dose was evaluated. If higher TKD or TKX doses or an additional opioid i.e., butorphanol or buprenorphine, was included, the resulting analgesia may be sufficient to provide a surgical anesthesia plane without additional isoflurane.

## 5. Conclusions

In summary, our findings indicate that general pig anesthesia induction with TKD is comparable to TKX when undergoing cryptorchid surgery for short-term (45-min) and long-term (90-min) anesthesia. TKD is a viable option to TKX in pigs. Like TKX, TKD provides a rapid onset of anesthetic induction, anesthetic maintenance, and recovery quality without significant alterations to duration or physiological parameters, yet requires the addition of isoflurane.

## Figures and Tables

**Table 1:** Time (min) presented as median ± interquartile range (IQR) after TKD or TKX administration until the confirmation of sternal recumbency, lateral recumbency, loss of withdrawal reflex, loss of jaw tone, and loss of palpebral reflex.

Observed Parameters (Induction Phase)	Time (min) after Anesthetic Drug Administration		*p*-Value
	TKD Group	TKX Group	
Sternal recumbency	1.14 (1.05–1.35)	1.04 (0.79–1.55)	0.970
Lateral recumbency	1.82 (1.49–2.36)	1.59 (1.40–2.16)	0.820
Loss of withdrawal reflex	2.90 (2.55–6.80)	3.68 (3.36–4.70)	0.597
Loss of jaw tone	5.04 (2.71–7.55)	4.03 (3.55–5.13)	0.880
Loss of palpebral reflex	5.75 (3.62–9.99)	4.85 (4.28–7.88)	0.821

TKD; combination of tiletamine/zolazepam, ketamine, and dexmedetomidine, TKX; combination of tiletamine/zolazepam, ketamine, and xylazine.

**Table 2:** Time (min) presented as median ± interquartile range (IQR) after atipamezole administration until the confirmation of the return of palpebral reflex, return of jaw tone, return of withdrawal reflex, return to sternal recumbency, and the ability to stand and walk following short-term (45-min) and long-term anesthesia (90-min) between the TKD and TKX groups.

Observed Parameters (Recovery Phase)	Time (min) after Atipamezole Administration					
	Short-Term Anesthesia (45-min)		*p*-Value	Long-Term Anesthesia (90-min)		*p*-Value
	TKD Group	TKX Group		TKD Group	TKX Group	
Return of palpebral reflex	3.0 (2.0–9.0)	7.0 (5.0–13.0)	0.347	2.0 (2.0–8.0)	15.0 (8.0–16.0)	0.138
Return of jaw tone	10.0 (3.0–14.0)	6.0 (6.0–8.0)	0.916	7.0 (3.0–9.0)	7.0 (7.0–10.0)	0.597
Return of withdrawal reflex	12.0 (7.0–24.0)	11.0 (6.0–19.0)	0.753	10.0 (3.0–14.0)	17.0 (12.0–20.0)	0.251
Return to sternal recumbency	18.0 (8.0–39.0)	35.0 (25.0–35.0)	0.600	18.0 (17.0–32.0)	28.0 (17.0–100.0)	0.530
Able to stand	84.0 (71.0–88.0)	81.0 (69.0–171.0)	0.917	39.0 (26.0–96.0)	147.0 (100.0–221.0)	0.117
Able to walk	84.0 (74.0–110.0)	87.0 (81.0–188.0)	0.754	58.0 (37.0–97.0)	149.0 (101.0–223.0)	0.175

TKD; combination of tiletamine/zolazepam, ketamine, and dexmedetomidine, TKX; combination of tiletamine/zolazepam, ketamine and xylazine.

**Table 3:** Average usage of isoflurane concentration, presented as median ± interquartile range (IQR), used throughout the anesthesia in TKD and TKX groups during short-term (45-min) and long-term anesthesia (90-min).

Observed Group	Average Usage of Isoflurane Concentration (%)		*p*-Value
	TKD Group	TKX Group	
Short-term anesthesia (45-min)	0.18 (0.18–0.65)	0.60 (0.33–1.00)	0.249
Long-term anesthesia (90-min)	0.45 (0.39–0.54)	0.16 (0.12–0.37)	0.151

TKD; combination of tiletamine/zolazepam, ketamine and dexmedetomidine, TKX; combination of tiletamine/zolazepam, ketamine and xylazine.

## Data Availability

The data presented in this study are available in this article. Further information is available upon request from the corresponding author.
